# Asymmetry in the Cytoarchitecture of the Area 44 Homolog of the Brain of the Chimpanzee *Pan troglodytes*

**DOI:** 10.3389/fnana.2020.00055

**Published:** 2020-08-21

**Authors:** Jean-Marie Graïc, Antonella Peruffo, Livio Corain, Cinzia Centelleghe, Alberto Granato, Emanuela Zanellato, Bruno Cozzi

**Affiliations:** ^1^Department of Comparative Biomedicine and Food Science, University of Padua, Padua, Italy; ^2^Department of Management and Engineering, University of Padua, Padua, Italy; ^3^Department of Psychology, Catholic University of the Sacred Heart, Milan, Italy

**Keywords:** asymmetry, area 44, Broca area, cytoarchitecture, pan troglodytes, cerebral cortex

## Abstract

The evolution of the brain in apes and man followed a joint pathway stemming from common ancestors 5–10 million years ago. However, although apparently sharing similar organization and neurochemical properties, association areas of the isocortex remain one of the cornerstones of what sets humans aside from other primates. Brodmann’s area 44, the area of Broca, is known for its implication in speech, and thus indirectly is a key mark of human uniqueness. This latero-caudal part of the frontal lobe shows a marked functional asymmetry in humans, and takes part in other complex functions, including learning and imitation, tool use, music and contains the mirror neuron system (MNS). Since the main features in the cytoarchitecture of Broca’s area remains relatively constant in hominids, including in our closest relative, the chimpanzee *Pan troglodytes*, investigations on the finer structure, cellular organization, connectivity and eventual asymmetry of area 44 have a direct bearing on the understanding of the neural mechanisms at the base of our language. The semi-automated image analysis technology that we employed in the current study showed that the structure of the cortical layers of the chimpanzee contains elements of asymmetry that are discussed in relation to the corresponding human areas and the putative resulting disparity of function.

## Introduction

Apes of the genus *Pan* parted from the human evolutionary path between 5 and 12 million years ago (Wakeley, 2008). Factually, the present-day chimpanzee (*Pan troglodytes*) and bonobo (*Pan paniscus*), are our closest relative species and with them we share many aspects of our anatomy and physiology. Much like the human brain, the chimpanzee isocortex is highly developed, and forms deep circumvolutions, increasing its cortical surface within the capacity of the cranial cavity, although several factors intervene (Striedter et al., 2015). The expansion of the cerebral surface implies a raise in absolute number of neurons, which offers greater computing power to process input, elaborate cognition, or greater precision and modulation (Chittka and Niven, 2009). The chimpanzee brain weighs about one third of the human brain, but the encephalization quotient ranks second among terrestrial mammals (Cozzi et al., 2016). The frontal lobe of the apes of the genus *Pan* contain wide frontal and prefrontal cortical areas, which in humans are devoted to higher functions including reasoning and development of the personality traits. The *pars opercularis* and *pars triangularis* are located at the back of the lower frontal gyrus of the left hemisphere of the human brain, and together form the Broca area, named after its discovery by Pierre Paul Broca (Broca, 1861). These two parts of the Broca area correspond to Brodmann’s areas 44 and 45, respectively, although it has been since broadened functionally (Amunts et al., 2010; Zilles and Amunts, 2018).

The area of Broca is a *functional* entity that corresponds to area 44 and 45 of the *left* hemisphere, associated with the control of language (Broca, 1861; Hervé, 1888; Penfield and Rasmussen, 1950; Roland, 1984, for a review see Nieuwenhuys et al., 2008) in most right-handers as well as left-handers (Branche et al., 1964), and to von Economo’s prerolandic region 8 (von Economo and Koskinas, 1925). Broca’s area has been a major subject in neurology history (Finger, 2005; Finger et al., 2009; Fingers et al., 2010), and the canonic concept implies that an obstruction of the left upper middle cerebral artery results in a stroke that affects the posterior region of the left frontal gyrus, the consequences of which include paralysis of the right side of the body and loss of speech (aphasia), whereas the identical process on the right upper branch of the middle cerebral artery does not impair speech in most cases. This has, however, been questioned several times in more recent years (Quiñones-Hinojosa et al., 2003; Keller et al., 2009a).

In addition to language processing (including production and comprehension), Brodmann’s area 44 has been shown to be involved in other higher cognitive functions including music, calculus and working memory (Fadiga et al., 2006, 2009; Hickok, 2009). Additionally, complex motor functions such as sign language (Horwitz et al., 2003; Emmorey, 2006), semantic gestures accompanying language (Skipper et al., 2007; Brown and Yuan, 2018), grabbing objects and even intelligent tool use (Hopkins et al., 2017) have been reported. In a recent study, Wakita (2014) detected via functional near-infrared spectroscopy (fNIRS) that area 44 was activated in situations of vicarious learning, i.e., careful observation of actions of another. This process involves the mirror neuron system (MNS) and has been studied in the area immediately caudal to the area 44, Brodmann’s area 6 in non-human primates (di Pellegrino et al., 1992; Rizzolatti et al., 1996; Welsh et al., 1996; Rizzolatti and Arbib, 1998; Buccino et al., 2001, 2004; Sundara et al., 2001; Nelissen et al., 2005; Nishitani et al., 2005; Skipper et al., 2007). Consistent with previous studies that confirmed the importance of the Broca area in the recognition and imitation of actions in humans (Buccino et al., 2004; Craighero et al., 2007), the results of Wakita (2014) suggest that area 44 is also implicated in the MNS. Finally, the Broca area also has important roles in local visual search (Fink et al., 2006) and in the planification and imagination of movement (Thoenissen et al., 2002; for a review see Ferrari and Rizzolatti, 2014). Some also proposed a dominance of phonological cues activating Broca’s area (Heim et al., 2008).

Since speech functions are lateralized to the left, the contralateral area 44 is therefore not part of Broca’s complex and has not been found to carry specific function. Interestingly, Brodmann’s area 22 (a.k.a. Wernicke’s area), which acts in synergy with Broca’s area for language, also shows a functional lateralization to the left (Parker et al., 2005). Consequently, there is general agreement over the fact that the anatomical asymmetry of human area 44 and 22 are at the root of the lateralization and consequent dominance of the language function of in one hemisphere (Geschwind and Levitsky, 1968; Galaburda et al., 1978a,b;Geschwind and Galaburda, 1985a,b,c; Galaburda, 1993). However, interhemispheric asymmetry studies report conflicting results, particularly in macroscopic studies (Toga and Thompson, 2003), while cytoarchitectural studies overall seem to support the existence of a structural asymmetry (Falzi et al., 1982; Amunts et al., 1999; Cantalupo and Hopkins, 2001). The cytoarchitecture of the Broca’s area was among the first described (Brodmann, 1909; Knauer, 1909; Vogt, 1910; Riegele, 1931), although the precise boundaries of Broca’s area are still uncertain despite a large body of research. Specifically, authors for decades have disagreed over either Brodmann’s area 44 was the sole representative of Broca’s area or included area 45, or even are 47 (see Uylings et al., 1999). It seems therefore that the location of Broca’s area and its topographical landmarks relative to major circumvolutions show frequent variations (Amunts et al., 1999).

Human areas 44 and 45 possess the six-layer organization common to other regions of the isocortex. However, area 44 is considered to be an agranular transition from area 6 to the granular area 45 (Amunts and Zilles, 2006). The presence of magnopyramidal neurons in layer 3 and 5 is also reported (Judas and Cepanec, 2007). Globally, areas 44 and 45 usually present anatomically similar features and hence are said to take part in related functions, even if activation and recording studies highlighted that electrical activity in area 44 or putative area 44 was circumscribed and specific to that region, and seemingly diverse from area 45 (Luppino et al., 1991; Petrides et al., 2005).

The question whether a functional equivalent of Broca’s area exists in other primates remains open, even more so when referring to our closer animal relatives, the apes belonging to the genus *Pan* (*Pan troglodytes* and *P. paniscus*). Stereotaxic atlases (Connolly, 1950; DeLucchi et al., 1965) and a series of published reports (Sperino, 1897; Buxhoeveden and Casanova, 2000; Cantalupo and Hopkins, 2001; Sherwood et al., 2003; Petrides and Pandya, 2004; Petrides, 2006) allow a topographic and cytoarchitectonic identification of an area of the brain of non-human primates (including *Pan troglodytes*) homolog to that of the human area 44, based on the limiting, albeit seemingly more variable on the left, inferior frontal and precentral sulci (Keller et al., 2009b, 2012; Amunts and Zilles, 2012; Zilles and Amunts, 2018), similarly to what has been proposed in lower monkeys (Galaburda and Pandya, 1982; Deacon, 1992; Preuss, 2000). However, the pattern of organization of the cytoarchitecture does not precisely follow the superficial landmarks listed above (Sherwood et al., 2003). The anatomical lateral asymmetry seems to be more disputed, since macroscopic differences were found in the *planum temporale* (Gannon et al., 1998) but seemingly not in the Broca area (Keller et al., 2009b, 2012). Lastly, few functional studies have been published (Taglialatela et al., 2006, 2008, 2011).

Assessing similarities and differences in the area 44 of our closest but speechless relative may improve our current understanding of the anatomical and physiological basis of spoken language. To this effect, recent reports denied the presence of cytoarchitectonic asymmetries in *Pan troglodytes* (Schenker et al., 2010). Our study of area 44 of *Pan troglodytes* is an attempt to (i) to ascertain the eventual presence of structural left-right asymmetries; (ii) assess whether the cellular structure of area 44 of the chimpanzee is similar to that of humans; and (iii) evaluate whether any eventual anatomical difference may hint at a difference in function.

## Materials and Methods

For the present work we used the brains of four adult chimpanzees (*Pan troglodytes*), whose anamnestic data is shown in Table 1. The apes were brought to the Department of Comparative Biomedicine and Food Science of the University of Padova for post-mortem examination. Causes of death were unrelated to the nervous system and macro- and microscopic examination of the brain did not identify lesions or anomalies.

**TABLE 1:** Information on the four chimpanzee (*Pan troglodytes*) specimen.

**ID**	**Sampling date**	**Sex**	**Age (years)**	**Weight (kg)**	**Cause of death**
36675	22.02.2011	M	adult	40 (est.)	Cardio-circulatory failure, possibly resulting from drowning
64361	19.05.2015	M	25	62.20	Cardiac arrest due to fibrous cardiomyopathy Severe enteric parasitosis as a co-factor of death.
68010	29.03.2016	F	40	40 (est.)	Multifactorial cause; systemic mycosis, purulent infection of tonsils and tongue may have led to endotoxicosis and breathing-difficulty condition
70113	05.10.2016	M	30	89.35	Heart and respiratory failure resulting from severe acute pancreatitis

### Tissue Processing

The brains were sampled during necropsy, and immediately immersed in 4% phosphate-buffered cold formalin for at least 1 month before trimming. In all animals, the post mortem interval was within 6 h.

**FIGURE 1:**
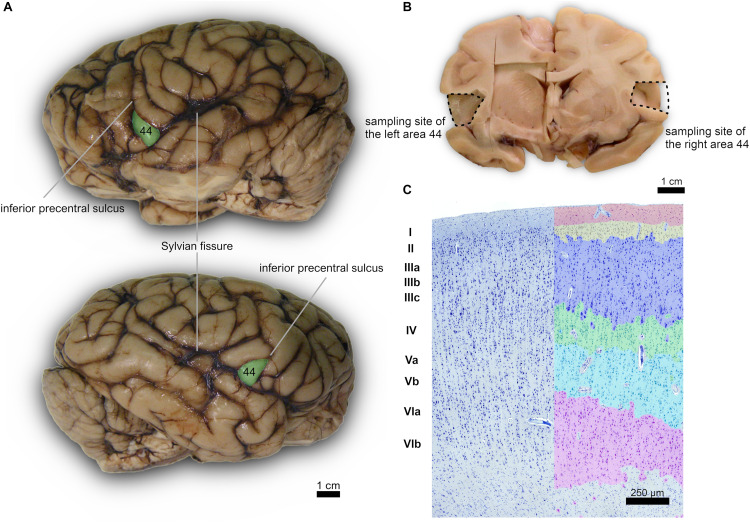
**(A)** Left (top) and right (bottom) lateral side of a *Pan troglodytes* cerebrum showing the area 44 of Brodmann; **(B)** Coronal section at the area 44 level showing the sampled site on each side; **(C)** Microphotographs of Nissl-stained cortical gray matter of *Pan troglodytes*; on the right, the stratigraphy of a digitized section colored with GIMP software: the cortical layers are distinguishable, from the outside to the inside, from the colors red (I layer), yellow (II layer), blue (III layer), green (IV layer), turquoise (V layer), pink (VI layer). On the left the same section without the colored demarcation of the six layers. Bar is 250 μm.

Subsequently, after a month in formalin, areas 44 from the left and right cerebral hemispheres were identified at the foot of the third frontal gyrus on each side and sampled (Figure 1). After trimming, the samples were processed for embedding in paraffin. Thin (8 μm-thick) paraffin sections were then obtained using a microtome and finally mounted on glass slides.

The same standard Nissl staining was performed for each section (*n* = 16), using the same solutions. Briefly, sections were passed in xylenes baths to remove paraffin, then in graded alcohols for hydration and washed in distilled water before a bath in a 0.4% thionine solution for 4 min. The sections were subsequently thoroughly washed in tap water before passing through graded alcohols for dehydration and subsequent xylene baths, before being covered using mounting medium and cover-slipping glasses. In all of these steps, all the tissues ran through the exact same procedures.

### Digital Analysis

Nissl stained sections were scanned using a semi-automated diagnostics digital microscope at 20x magnification (D-Sight 2.0, Menarini Diagnostics, Florence, Italy) using the same settings. Output images were then opened in a raster image editor (GNU Image Manipulation Program “GIMP,” Free Software Foundation, Inc.) to identify and segment each cortical layer, following the accepted cortical features (below).

According to Zilles and Amunts (2012, 2018), the cerebral cortex in the area 44 of the chimpanzee is deemed to have 6 distinguishable layers, as in the rest of mammals most generally. The first layer (molecular layer) mostly devoid of neuron bodies but rich in dendrites and axons from the deeper layers and connecting areas, lies most externally. The external granular layer constitutes layer 2, highly dense in small to medium pyramidal cells, with its lower margin merging with the third layer. The external pyramidal layer is characterized by medium-small pyramidal cells and stellate cells, mostly in its upper part, while the lower part contains larger and intensely stained pyramidal neurons. Below is the fourth or internal granular layer, usually rich in small granular neurons. In the case of area 44, is it “dysgranular,” with scant granular neurons in a reduced layer 4. The internal pyramidal layer (5) is characterized by medium to large pyramidal neurons, with fewer neurons in the lower band. The innermost layer 6 contains fusiform neurons orientated longitudinally and is usually wide and interspersed with large myelin sheath groups.

**FIGURE 2:**
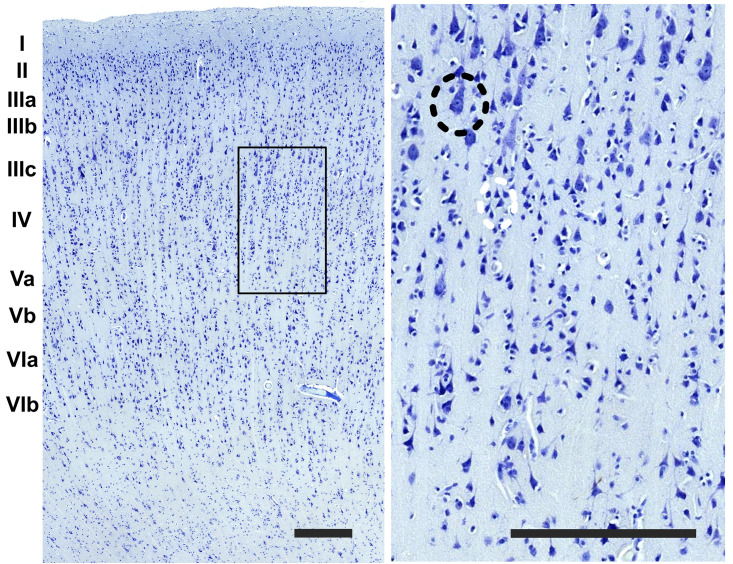
Detail of the image in the boundary between the layer III and IV (rectangle). On the right image upper left side, a pyramidal cell of the third layer (black dashed circle) and a grouping of small interneurons of the fourth layer (white dashed circle). Bar is 250 μm.

The cerebral cortex of the area 44 was subdivided into layers by three independent neuroanatomists and a student (BC, J-MG, AP, and EZ), only one of which (EZ) knew about the hemispheric side of each section, all according to the precepts above. Six layers were individualized without attempting to subdivide them (Figures 1, 2). Large and middle-sized vessels were excluded when possible from the layers to avoid artifacts.

The resulting layers were analyzed separately using a custom image analysis algorithm in Matlab (The MathWorks, Inc.) using local space-varying threshold (Poletti et al., 2012) applied to the image to separate the background and the local density of the foreground objects (mainly cells), resulting in a separation of the densest (possibly including clustered and cluttered cells) and most sparse regions (for additional details see Grisan et al., 2018; Peruffo et al., 2019). Multiple thresholds were implemented identically to each layer to separate the cells from the background matrix, and the particle analysis was ranged from 25 to 400 μm^2^ to avoid glial cells as well as large artifacts. The cells identified were then characterized along morphometric descriptors.

The output tables comprising tens of thousands of cells were analyzed statistically.

For each layer, five cell morphometric descriptors were measured: area, perimeter, major axis length, minor axis length (values were measured in μm or μm_2_) and density (number of neighboring cells counted within a radius of 50 μm all around a given cell).

For the statistical analysis four parameters were analyzed: area, perimeter, aspect ratio (AR) and density.

Aspect Ratio is a parameter that measures the regularity of the shape of the cell. It is the ratio of the major and minor axes of the smallest ellipse that fits the cell within it. It is calculated by the formula:

$$A\,R=\frac{M\,a\,j\,o\,r\,A\,x\,i\,s\,l\,e\,n\,g\,t\,h}{M\,i\,n\,o\,r\,A\,x\,i\,s\,l\,e\,n\,g\,t\,h}$$

A low ratio indicates that the cell inscribed in the ellipse is more regular in shape than a high ratio, indicating that the cell shape is more irregular. The greater the ratio of the axes, the greater the ellipse that describes the cell and, consequently, the more irregular the form of the cell.

The density was defined here as a surface density, by the number of neighboring cells of a given cell within a 50 (μm radius.

### Statistical Analysis

A two-sided ANOVA analysis was performed, first by applying a one-way factor analysis for (a) hemispheric side (regardless of the cortical layer) and (b) layer (regardless of the hemispheric side). Secondly, to test the interaction effect, a two-way ANOVA analysis of (c) both side and layer (for different parameters) was performed. A *p*-value inferior to 0.05 was considered a significant change attributable to the factors (a), (b), or (c).

Separately for each of the five parameters, i.e., Y = (area, perimeter, AR density), has been formalized the following statistical linear model:

$$Y_{i\,j\,k}=\mu +\tau_{i}+\beta_{j}+{\left(\tau \,\beta \right)}_{i\,j}+\varepsilon_{i\,j\,k}$$

where τ represents the layer τ_*I*_ = (L1, L2, L3, L4, L5, L6), β represents the side β_*j*_ = (right, left) and (τβ) represents the 6 ×2 = 12 layer-side interactions. Finally, we assumed e as a normal distribution.

## Results

### Macroscopic Anatomy

A certain individual variability in the precise localization of secondary sulci was seen. However, for each of the four specimen we noted a subtle left-right asymmetry. In particular, the left *pars triangularis* corresponding to the area 45 was relatively easily found on the inferior frontal gyrus, once the landmark of the inferior precentral sulcus was found along the caudo-lateral part of the frontal lobe, down toward the Sylvian sulcus (Figures 1A,B). The area 44 was found caudally relatively to this *pars triangularis*, in the *pars opercularis*. We did not attempt to find the confines of one or the other region histologically.

### Microscopic Anatomy

The organization of the cortex showed a relatively thin layer 1, a layer 2-3 difficultly parted, with slightly higher density in layer 2, a relatively large granularity in layer 4 (Figures 1C, 2). Large pyramidal neurons could be clearly seen, unevenly distributed along the lower band of layer 3, marking the upper border of layer 4, which was relatively thin and with granule neurons, sometimes in clusters. Large pyramidal neurons were also found in lower layer 5, while the upper part was less dense. Layer 6 was more populated than layer 5 with a neat border with white matter. Clear columns could be seen along most of the thickness of the cortex and large spaces between those columns marked important myelin tracts in the infragranular layers (Figure 2). The general picture is that of a dysgranuar cortex, with the irregular presence of a fourth layer, closely bordered by large pyramidal neurons externally.

### Results of the Morphometric Data Analysis

#### Area

**FIGURE 3:**
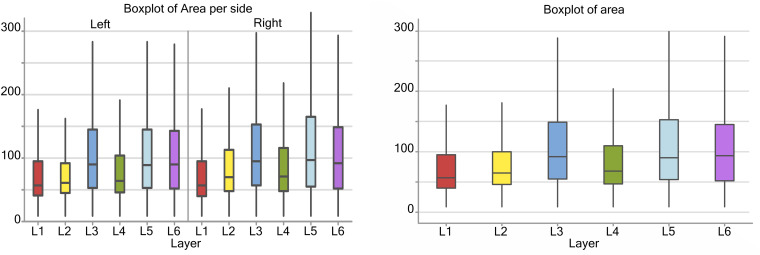
Boxplots of the cell area of L1 (red), L2 (yellow), L3 (blue), L4 (green), L5 (light blue), L6 (purple). In the left boxplot, the area per layer was evaluated without considering side of the hemisphere. In the right boxplot, the area per layer was evaluated by side of cerebral hemisphere. Area was measured in μm^2^.

Considering the cortical layers of the area 44, larger neurons were found in layer L3, L5, and L6, most usually associated with pyramidal neurons, with a mean area over 75 μm^2^, while L1, L2, and L4 had average cell areas closer to 50–60 μm^2^ (Figure 3). When accounting for hemispheric side, slight differences between left and right could be seen. Neurons on the left side were generally smaller in area, especially in L1, L2, and L4. The medians for the area shifted to smaller values on the left, which implies that the distribution is centered on lower values and is notably more unevenly distributed toward larger values (positive skew). Neurons with the largest area were found in right L3 and L5 (280 and 290 μm^2^ respectively).

**FIGURE 4:**
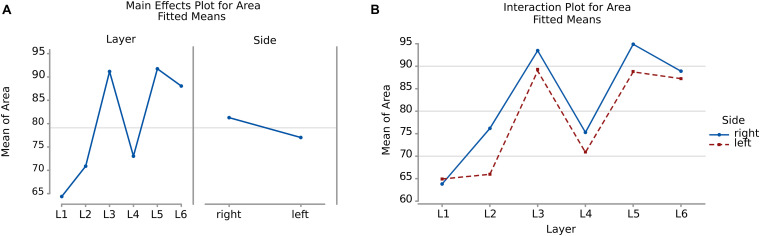
**(A)** To the left is the main effect plot showing the cell area variation depending on the layer. On the right is the main effect plot displaying cell area variation in the right and left-brain hemispheres. **(B)** Interaction plot showing cell area variation in the combination effect of the factor layer and side (right side blue line, left side red line).

There was a significant difference (*p* < 0.001, Table 2) between the average areas considering layer of origin and brain side factors separately (Figure 4A) as well as the layer-side interaction (*p* < 0.001, Table 2 and Figure 4B). The former shows that each layer harbors cell populations strongly different in size (Table 3). The latter means that the overall difference of average area between the two hemispheres varies by layer. Neurons in the left hemisphere tended to be smaller than those in the right hemisphere (*p* < 0.001).

**TABLE 2:** *p*-values separately for each factor (a and b) and their interaction (c) for the morphological parameter cell area (stand deviation 0.66).

**Area**	**Factor**	***p*-Value**
	(a) Layer	<0.001
	(b) Side	<0.001
	(c) Layer-Side	<0.001

**TABLE 3:** Numerical data ± standard error mean, Q1 and Q3 (first and third quartile), for the average area of neurons considering their layer (left), and their hemispheric side (right) and the average area of cells belonging to a given layer in a given hemisphere (layer and side).

***Mean area* (μ*m^2^)***							
***Per layer***		***Per side***		***Per layer and side***			
***L***	*81.6* ± *0.7*	***Left hemisph.***	*101* ± *0.2*	***L*_1 – LEFT_**	*82.9* ± *1.1*	***L*_1 – RIGHT_**	*80.6* ± *0.9*
***_1_***	(*Q1* = *40; Q3* = *95)*		(*Q1* = *49; Q3* = *132)*		(*Q1* = *41; Q3* = *95)*		(*Q1* = *40; Q3* = *95)*
***L***	*84.5* ± *0.5*			***L*_2 – LEFT_**	*77.5* ± *0.5*	***L*_2 – RIGHT_**	*93.8* ± *0.9*
***_2_***	(*Q1* = *46; Q3* = *100)*				(*Q1* = *45; Q3* = *93)*		(*Q1* = *48; Q3* = *113)*
***L***	*113* ± *0.3*			***L*_3 – LEFT_**	*111* ± *0.5*	***L*_3 – RIGHT_**	*115* ± *0.5*
***_3_***	(*Q1* = *55; Q3* = *149)*				(*Q1* = *53; Q3* = *145)*		(*Q1* = *57; Q3* = *153)*
***L***	*88.5* ± *0.4*	***Right hemisph.***	*107* ± *0.2*	***L*_4 – LEFT_**	*85.7* ± *0.5*	***L*_4 – RIGHT_**	*91.8* ± *0.6*
***_4_***	(*Q1* = *47; Q3* = *110)*		(*Q1* = *51; Q3* = *143)*		(*Q1* = *46; Q3* = *104)*		(*Q1* = *48; Q3* = *116)*
***L***	*114* ± *0.4*			***L*_5 – LEFT_**	*110* ± *0.6*	***L*_5 – RIGHT_**	*119* ± *0.7*
***_5_***	(*Q1* = *54; Q3* = *153)*				(*Q1* = *53; Q3* = *145)*		(*Q1* = *55; Q3* = *165)*
***L***	*108* ± *0.4*			***L*_6 – LEFT_**	*107* ± *0.6*	***L*_6 – RIGHT_**	*110* ± *0.6*
***_6_***	(*Q1* = *52; Q3* = *145)*				(*Q1* = *52; Q3* = *143)*		(*Q1* = *52; Q3* = *148)*

As shown by the significant interaction (*p* < 0.001), despite the area differences had similar patterns and were consistent between the same layers from each side, the neurons of the external granular and internal pyramidal layers were noticeably smaller on the left than on the right.

#### Perimeter

There was a positive correlation between area and perimeter: larger neurons tend to have a larger perimeter.

**FIGURE 5:**
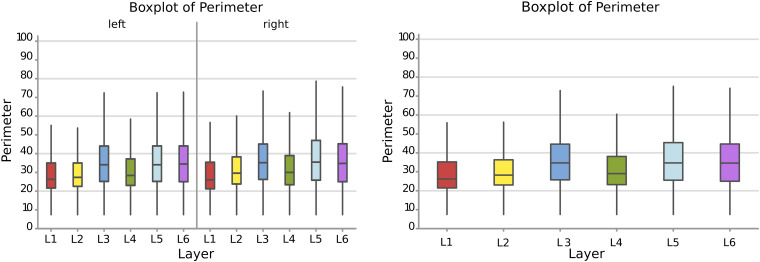
Boxplots comparing the perimeter of cells in L1 (blue), L2 (red), L3 (yellow), L4 (green), L5 (orange), L6 (light blue). In the left boxplot, the perimeter per layer is shown without considering the side of the hemisphere. In the right boxplot, the perimeter per layer was evaluated by side of cerebral hemisphere. Perimeter was measured in μm.

Therefore, following the area, a larger perimeter was found in neurons of the layer L3, L5, and L6, with a mean perimeter over 36.8 μm, while L1, L2, and L4 had average cell perimeters closer to 30 – 32 μm (Figure 5). When accounting for side of the hemisphere, neurons on the left side were generally smaller in perimeter, especially in L1, L2, and L4. Neurons with the largest perimeter were found in right L3 and L5 (37.2 and 37.6 μm respectively). The fusiform cells of the 6th layer (especially on the left) showed a high perimeter/area ratio, which is consistent with the nature of their shape.

**FIGURE 6:**
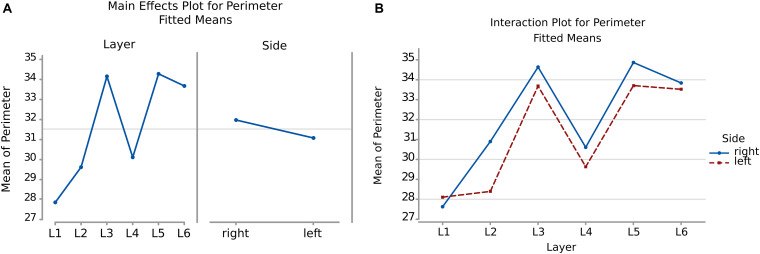
**(A)** To the left is the main effect plot showing the cell perimeter variation depending on the layer. On the right is the perimeter of all the cells in the left and right brain hemispheres. **(B)** Interaction plot showing the perimeter variation in the combination effect of the factor layer and side (right side blue line, left side red line).

The perimeter measurements were consistent with those of the area. Again, the *p*-values and the graphs show that there is a significant difference between the average perimeters, both considering the layer of origin, the side and the layer-side interaction (all *p* < 0.001, Table 4). However, layers 2 and 5 showed the largest difference (Figure 6B, Table 5) between sides, with larger average perimeters on the right side. Consequently, the trend of the graph in Figure 6B is similar to that of the area in Figure 4B.

**TABLE 4:** *p*-values separately for each factor (a and b) and their interaction (c) for the morphological parameters perimeter (stand deviation 0.03).

**Perimeter**	**Factor**	***p*-Value**
	(a) Layer	<0.001
	(b) Side	<0.001
	(c) Layer-Side	<0.001

**TABLE 5:** Numerical data ± standard error mean, Q1 and Q3 (first and third quartile), for the average perimeter of neurons considering their layer of origin (left), and their hemispheric side (right) and the average perimeter of cells belonging to a given layer for each hemisphere (layer by side).

***Average perimeter* (μ*m)***							
***Per layer***		***Per side***		***Per layer and side***			
***L*_1_**	*30.0* ± *0.1*	***Left hemisph.***	*34.4* ± *0.1*	***L*_1 – LEFT_**	*30.2* ± *0.2*	***L*_1– RIGHT_**	*29.8* ± *0.2*
	(*Q1* = *21; Q3* = *35)*		(*Q1* = *23; Q3* = *42)*		(*Q1* = *21; Q3* = *35)*		(*Q1* = *21; Q3* = *35)*
***L*_2_**	*31.3* ± *0.1*			***L*_2 – LEFT_**	*29.9* ± *0.1*	***L*_2 – RIGHT_**	*33.1* ± *0.1*
	(*Q1* = *23; Q3* = *36)*				(*Q1* = *22; Q3* = *35)*		(*Q1* = *23; Q3* = *38)*
***L*_3_**	*36.7* ± *0.1*			***L*_3 – LEFT_**	*36.1* ± *0.1*	***L*_3 – RIGHT_**	*37.1* ± *0.1*
	(*Q1* = *25; Q3* = *44)*				(*Q1* = *25; Q3* = *44)*		(*Q1* = *26; Q3* = *45)*
***L*_4_**	*32.0* ± *0.1*	***Right hemisph.***	*34.4* ± *0.1*	***L*_4 – LEFT_**	*31.5* ± *0.1*	***L*_4 – RIGHT_**	*32.6* ± *0.1*
	(*Q1* = *23; Q3* = *38)*		(*Q1* = *23; Q3* = *42)*		(*Q1* = *23; Q3* = *37)*		(*Q1* = *23; Q3* = *38)*
***L*_5_**	*36.8* ± *0.1*			***L*_5 – LEFT_**	*36.2* ± *0.1*	***L*_5 – RIGHT_**	*37.6* ± *0.1*
	(*Q1* = *25; Q3* = *45)*				(*Q1* = *25; Q3* = *44)*		(*Q1* = *25; Q3* = *47)*
***L*_6_**	*36.1* ± *0.0*			***L*_6 – LEFT_**	*35.9* ± *0.1*	***L*_6 – RIGHT_**	*36.3* ± *0.1*
	(*Q1* = *25; Q3* = *44)*				(*Q1* = *25; Q3* = *44)*		(*Q1* = *25; Q3* = *45)*

#### Aspect Ratio

**FIGURE 7:**
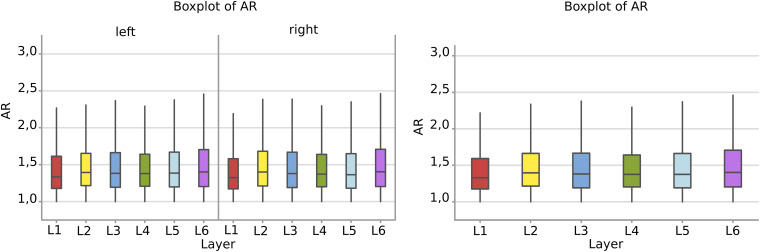
Boxplots comparing the AR of cells in L1 (red), L2 (yellow), L3 (blue), L4 (green), L5 (light blue), L6 (purple). In the left boxplot, the AR per layer is shown without considering side of the hemisphere. The median Aspect Ratio value of the cells is represented for each layer by the horizontal line in the colored rectangle. AR is a ratio of length and has no unit.

There was a remarkable similarity among layers which was not the case of perimeter and area. The most irregular cells were in the sixth layer, with a mean AR over 1,49 where most of the large pyramidal neurons were found, with no difference between the right and left hemisphere (Figure 7).

**FIGURE 8:**
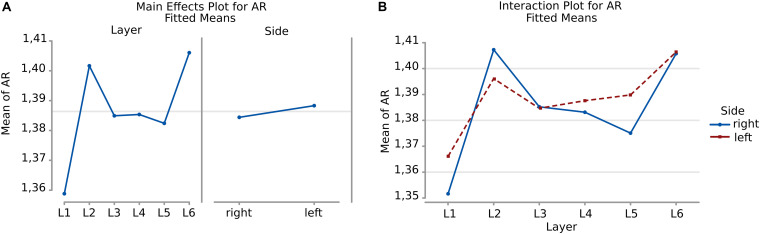
**(A)** To the left is the main effect plot showing the cell AR variation depending on the layer. On the right, the main effect plot displaying cell AR variation in the right and left-brain hemispheres. **(B)** Interaction plot showing cell AR variation in the combination effect of the factor layer and side (right side blue line, left side red line).

Neurons appeared more irregular to the left than to the right (*p* = 0.031, Table 6, and Figure 8A), and layers also resulted significantly affecting AR (*p* < 0.001, Table 6). The significant interaction (*p* < 0.001) implies that AR differences had similar patterns and were consistent between the same layers from each side (Table 7). The most irregular neuronal cells were included in layer 2 and 6 (Figures 8A,B). Interestingly, the AR of the neurons in L2 and L5 appeared the most different across sides, but inversely. Neurons of the left L2 (outer granular layer) appeared more regular than the right side, while ones from the left L5 (inner pyramidal layer) appeared more irregular than in the right hemisphere.

**TABLE 6:** AR *p*-values separately for each factor (a and b) and their interaction (c) for the morphological parameter AR (stand deviation 0.17).

**Aspect Ratio**	**Factor**	***p-*Value**
	(a) Layer	<0.001
	(b) Side	0.031
	(c) Layer-Side	<0.001

**TABLE 7:** 2 ± standard error mean, Q1 and Q3 (first and third quartile), for the average AR of neurons considering their layer (left), and their hemispheric side (right) and the average AR of cells belonging to a given layer for each hemisphere (layer by side).

***Medium AR***							
***Per layer***		***Per side***		***Per layer and side***			
***L*_1_**	*1.4* ± *0.003*	***Left hemisph.***	*1.5* ± *0.001*	***L*_1 – LEFT_**	*1.4* ± *0.006*	***L*_1 – RIGHT_**	*1.4* ± *0.005*
	(*Q1* = *1.1; Q3* = *1.5)*		(*Q1* = *1.1; Q3* = *1.6)*		(*Q1* = *1.1; Q3* = *1.6)*		(*Q1* = *1.1; Q3* = *1.5)*
***L*_2_**	*1.5* ± *0.002*			***L*_2 – LEFT_**	*1.5* ± *0.003*	***L*_2 – RIGHT_**	*1.5* ± *0.004*
	(*Q1* = *1.2; Q3* = *1.6)*				(*Q1* = *1.2; Q3* = *1.6)*		(*Q1* = *1.2; Q3* = *1.6)*
***L*_3_**	*1.5* ± *0.001*			***L*_3 – LEFT_**	*1.5* ± *0.002*	***L*_3 – RIGHT_**	*1.5* ± *0.002*
	(*Q1* = *1.1; Q3* = *1.6)*				(*Q1* = *1.1; Q3* = *1.6)*		(*Q1* = *1.1; Q3* = *1.6)*
***L*_4_**	*1.5* ± *0.002*	***Right hemisph.***	*1.5* ± *0.001*	***L*_4 – LEFT_**	*1.5* ± *0.003*	***L*_4 – RIGHT_**	*1.5* ± *0.003*
	(*Q1* = *1.2; Q3* = *1.6)*		(*Q1* = *1.1; Q3* = *1.6)*		(*Q1* = *1.2; Q3* = *1.6)*		(*Q1* = *1.2; Q3* = *1.6)*
***L*_5_**	*1.5* ± *0.002*			***L*_5 – LEFT_**	*1.5* ± *0.002*	***L*_5 – RIGHT_**	*1.5* ± *0.003*
	(*Q1* = *1.1; Q3* = *1.6)*				(*Q1* = *1.1; Q3* = *1.6)*		(*Q1* = *1.1; Q3* = *1.6)*
***L*_6_**	*1.5* ± *0.002*			***L*_6 – LEFT_**	*1.5* ± *0.003*	***L*_6 – RIGHT_**	*1.5* ± *0.003*
	(*Q1* = *1.2; Q3* = *1.7)*				(*Q1* = *1.2; Q3* = *1.7)*		(*Q1* = *1.2; Q3* = *1.7)*

#### Surface Density

**FIGURE 9:**
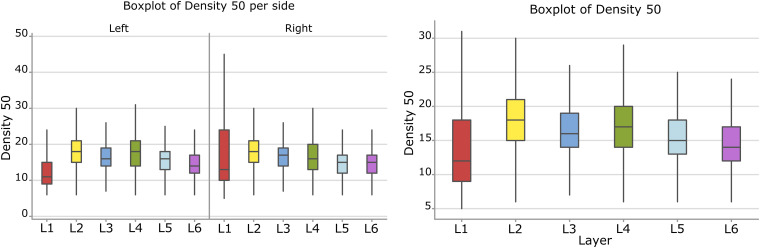
Boxplots comparing the density of cells in L1 (blue), L2 (red), L3 (yellow), L4 (green), L5 (orange), L6 (light blue). In the left boxplot, the perimeter per layer is shown without considering the side of the hemisphere. In the right boxplot, the density per layer was evaluated by side of cerebral hemisphere. Density was measured in cells number in a 50 μm radius.

Considering the layers for the descriptor density, the densest neural cells were found in the L2 (outer granular layers) with a mean density over 18.3 cells within a 50 μm radius (Figure 9). When accounting for side, neurons on the right side were overall denser. The layer L1 showed the smallest density but only for the left hemisphere with a mean density of 12.3 cells within a 50 μm radius (Figure 9 and Table 8).

**TABLE 8:** Density *p*-values separately for each factor (a and b) and their interaction (c) for the parametersdensity (stand deviation 0.03).

**Density**	**Factor**	***p*-Value**
	(a) Layer	<0.001
	(b) Side	<0.001
	(c) Layer-Side	<0.001

**FIGURE 10:**
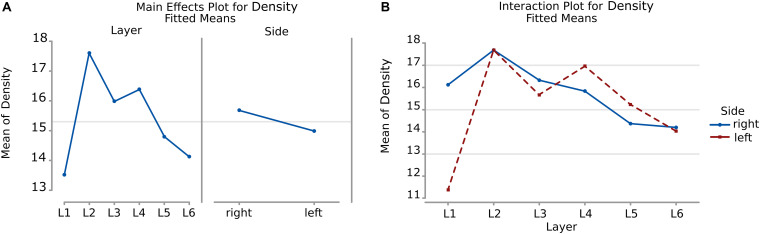
**(A)** To the left is the main effect plot showing the cell density variation depending on the layer. On the right is the main effect plot displaying cell density variation in the right and left-brain hemispheres. **(B)** Interaction plot showing cell density variation in the combination effect of the factor layer and side (right side blue line, left side red line).

The density of cells detected by μm_2_ was significantly different considering alternatively layer, side or their interaction (a, b, and c, *p* < 0.001, Table 8). Cells in L2 and L4 were notably the densest, while L1 and L6 were the least dense (Figure 10A, Table 9). Area 44 neurons of the left hemisphere appeared less dense than on the right hemisphere. On the contrary, the L4 (inner granular layer) neurons on the left side were denser than on the right (Figure 10B) while L1 was much less dense in the left hemisphere than in the right hemisphere.

**TABLE 9:** Numerical data ± standard error mean, Q1 and Q3 (first and third quartile), for the average density of neurons considering their layer (left), and their hemispheric side (right) and the average density belonging to a given layer for each hemisphere (layer by side).

***Average density (number of cells in a 50* μ*m radius)***							
***Per layer***		***Per side***		***Per layer and side***			
***L*_1_**	*18.1* ± *0.16*	***Left hemisph.***	*16.1* ± *0.01*	***L*_1 – LEFT_**	*12.4* ± *0.08*	***L*_1 – RIGHT_**	*22.1* ± *0.27*
	(*Q1* = *9; Q3* = *18)*		(*Q1* = *13; Q3* = *19)*		(*Q1* = *9; Q3* = *15)*		(*Q1* = *10; Q3* = *24)*
***L*_2_**	*18.3* ± *0.04*			***L*_2 – LEFT_**	*18.2* ± *0.05*	***L*_2 – RIGHT_**	*18.4* ± *0.06*
	(*Q1* = *15; Q3* = *21)*				(*Q1* = *15; Q3* = *21)*		(*Q1* = *15; Q3* = *21)*
***L*_3_**	*16.5* ± *0.01*			***L*_3 – LEFT_**	*16.1* ± *0.02*	***L*_3 – RIGHT_**	*16.8* ± *0.02*
	(*Q1* = *14; Q3* = *19)*				(*Q1* = *14; Q3* = *19)*		(*Q1* = *14; Q3* = *19)*
***L*_4_**	*17.1* ± *0.03*	***Right hemisph.***	*16.6* ± *0.02*	***L*_4 – LEFT_**	*17.6* ± *0.04*	***L*_4 – RIGHT_**	*16.6* ± *0.04*
	(*Q1* = *14; Q3* = *20)*		(*Q1* = *13; Q3* = *19)*		(*Q1* = *14; Q3* = *21)*		(*Q1* = *13; Q3* = *20)*
***L*_5_**	*15.3* ± *0.02*			***L*_5 – LEFT_**	*15.7* ± *0.03*	***L*_5 – RIGHT_**	*14.8* ± *0.03*
	(*Q1* = *13; Q3* = *18)*				(*Q1* = *13; Q3* = *18)*		(*Q1* = *12; Q3* = *17)*
***L*_6_**	*14.6* ± *0.02*			***L*_6 – LEFT_**	*14.5* ± *0.03*	***L*_6 – RIGHT_**	*14.6* ± *0.03*
	(*Q1* = *12; Q3* = *17)*				(*Q1* = *12; Q3* = *17)*		(*Q1* = *12; Q3* = *17)*

## Discussion

The brains of the four chimpanzees of our sampling series looked very similar for volume, shape and surface configurations of the major gyri (Figure 1). However, more subtle variations were present, including also minor discrepancies of configuration and shape of the gyri between the left and the right side, as should be expected in most normal mammalian brains. Macroscopic variations in the forking of sulci or cirumvolution thickness have been previously reported between left and right sides of the human area 44 (Sherwood et al., 2003; Keller et al., 2009b; Zilles and Amunts, 2018). The cortical gyri acquire their shape during brain development and their variability influence the shape and volume of the whole lower frontal gyrus in humans and non-human primates (Cantalupo and Hopkins, 2001). Magnetic resonance studies showed that humans and chimpanzee share a similar gross morphology of Broca’s area, including the presence of individual variations in shape and boundaries (Cantalupo and Hopkins, 2001; Keller et al., 2009b, 2012; Gómez-Robles et al., 2013), although recent data challenged this view (Rilling and Van Den Heuvel, 2018; Xiang et al., 2018, 2019). The limits of area 44 and 45 are not constant, and vary in shape, length, continuity and number of the isocortical sulci (Foundas et al., 1996, 1998; Tomaiuolo et al., 1999; Keller et al., 2007). Chimpanzees (but not macaques) show tendency to hand preference in certain manual activities (Hopkins and Leavens, 1998; Hopkins et al., 2004, 2005; Papademetriou et al., 2005; Fitch and Braccini, 2013), a fact emphasized by elusive hemispheric asymmetries in differential gene expression (Muntané et al., 2017), but unfortunately no data about handedness was available for the specimens of our group. Moreover, connectivity of areas 44 and 45 is similar in humans and monkeys (Kelly et al., 2010), although some specific tracts (i.e., the arcuate fasciculus and dorsal longitudinal tracts in general) may be less robust at least in monkeys, with potential consequences for language expression (Rilling et al., 2008; Eichert et al., 2018; Mars et al., 2019; Barrett et al., 2020). Differences in brain structure among apes are not so well documented and may be apparently related to the size of the striatum, cerebellum and hippocampus (Sherwood et al., 2004).

Architectonic and physiological studies have shown large similarities with the human area 44 in macaque monkeys, with a part devoted to orofacial muscles and thought to be premises to Broca’s area in humans (Petrides et al., 2005; Petrides, 2006). To our knowledge, it was first located and described as macroscopically asymmetrical in the chimpanzee by Sperino (1897), but has since been investigated further (Gannon et al., 1998; Sherwood et al., 2003; Schenker et al., 2010; Zilles and Amunts, 2018). Electrophysiological stimulation of the chimpanzee area homologous to the human Broca’s area induced movements of the vocal cords (FCBm area of Bailey et al., 1950). Notably it was observed that sulcal/gyral variations were akin to that of humans in the area (Schenker et al., 2010). The cortico-cortical connections around the arcuate sulcus also seem to show similarities to connection patterns in the human Broca area (Deacon, 1992).

In the present study, microscopic analysis of the Nissl-stained sections revealed the expected “dysgranular” six-layered organization of the cortex, with neither immediate appreciable asymmetry nor difference among the individual specimens. The cytoarchitecture of the area 44 of chimpanzee was remarkably similar to that of the human, corresponding to previous studies in monkeys (Sherwood et al., 2003; Petrides and Pandya, 2004; Zilles and Amunts, 2018). Mean area by layer (Figure 4A) as well as perimeter (Figure 6A) showed that, after a very small cell- and scarcely populated molecular layer, there was a clear alternation of granular (external and internal) and pyramidal (external and internal) layers, with remarkably similar sizes, larger for pyramidal layers (around 114 μm2 and 36 μm respectively) and smaller for granular layers (around 85 μm2 and 30 μm respectively). A consistent positive correlation between area and perimeter of the neurons pointed out that neurons with a larger perimeter also had a larger area and therefore were not excessively elongated. This was confirmed also by the morphological parameters related to shape, such as AR.

Comparison between sides for each layer (Figures 4A, 6A) showed that neurons of layers 2–6 of the left area 44 were consistently smaller than the corresponding elements of the right side, except for those of layer 1 (Tables 3, 5). The differences in area and perimeter were noticeable and constant (see Tables 2–5 for actual values and significance). Differences were even more noticeable in the left layer 2 (Figure 4B). The presence of large pyramidal neurons in the fifth layer of inner pyramidal layer was marked by larger cells (Figures 4B, 7), but more irregular and denser on the left side (Figures 8B, 10B). Recent findings confirmed the importance of upper-layer neurons in the differentiation between primates and other mammals (Charvet et al., 2017).

Interestingly, the second layer seemed to contain smaller, overall round neurons (Figures 4B, 6B, 8B). This reduced size is consistent with the increased presence on the left side of a group of small neurons (most likely granular cells). We have no way to establish whether this situation could be related to the numerosity of thalamo-recipient elements (additional to the canonic granules of layer 4, Peruffo et al., 2019) in the left area 44, a situation that would allow theoretically higher computational capabilities or superior functions. Here we emphasize that the density of cells in the inner granular layer was also sensibly higher in the left area 44 (Figure 10B).

Several studies documented cytoarchitectural differences between hemispheres and among ape species (Toga and Thompson, 2003). Buxhoeveden et al. (2001) developed a computerized imaging program to examine minicolumns in Nissl-stained slides of human, chimpanzee, and rhesus monkey planum temporale. They revealed that wider columns and more neuropil space on the left side existed in humans, but this asymmetry was absent in the chimpanzee and rhesus monkey (Buxhoeveden et al., 2001). Later, it was demonstrated that human regions of the prefrontal cortex have a significantly higher neuropil fraction than the other areas, in which the neuropil fraction was used as a proxy for global connectivity (Spocter et al., 2012). Interestingly, again, this difference was not found in chimpanzees’ prefrontal regions, which supports the conclusion that increase connectivity in the prefrontal cortex accompanied the evolution of the human brain.

Asymmetry in chimpanzee brain was found in the motor cortex (Sherwood et al., 2007). Analyzing the region of hand representation, they showed a leftward bias for higher layer II/III neuron density. However, there was no asymmetry in the fraction of Nissl-stained cell bodies (Sherwood et al., 2007). Several studies have in particular examined asymmetries in the cytoarchitectonic regions corresponding to Broca’s and Wernicke’s areas in humans and chimpanzees arguing globally for a continuity in the behavioral and brain asymmetries in chimpanzees and humans, quantitatively (Hopkins, 2013). In particular, the Broca area was characterized and subdivided by receptor type into subregions, one of which, M2 cholinergic receptors, showing a lateralization in the area 44 v + d (Amunts et al., 2010; Zilles and Palomero-Gallagher, 2017). Recently, Palomero-Gallagher and Zilles (2018) identified microstructural differences in proportions of neuropil volume and cell bodies in areas 44 and 45 of human, bonobo, chimpanzee, gorilla, orangutan, and macaque brains. Their results highlighted three main clusters: *Homo sapiens* has the largest neuropil proportion, great apes a markedly lower one and the macaque had the lowest, confirming previous general studies (Spocter et al., 2012).

Previous studies found leftward asymmetries in the human area 44, including magnopyramidal neuron size (Galaburda, 1980), volume (Amunts and Zilles, 2006), or neuron count (Uylings et al., 2005), even if not all of the differences were significant (Witelson and Kigar, 1988). Scheibel et al. (1985) reported a higher dendritic branching in the area 44 of the left hemisphere, although in most cases area 45 was separated, and for both regions, results of asymmetry were not uniform (Hayes and Lewis, 1996; Bogolepova and Malofeeva, 2001; Amunts et al., 2003; García et al., 2004; Uylings et al., 2005), but globally pointed toward a leftward asymmetry, contrarily to most volume studies (see below). Therefore, there seems to be broad cytoarchitectural indication of leftward asymmetry from at least Brodmann’s area 44, and possibly more variations in males (DeCasien et al., 2020). The hypothesis of a preexisting substrate in chimpanzees for Broca’s area development in humans is not entirely new, since asymmetry in the planum temporale has been put forward (Gannon et al., 1998). The differences evidenced by the present study support the existence of a structural asymmetry of the area 44 of the chimpanzee brain, due to the presence of smaller neurons on the left hemisphere, possibly a hint of enhanced lateralized input and integration, which do not support works singling out human cytoarchitecture (Spocter et al., 2012).

Recent studies (Bianchi et al., 2013) proved that humans and chimpanzees (but not macaques) share certain characteristics of neural growth, including relatively extended maturation of neurons and synapses into the mid-juvenile phase, a fact linked to the development of sociality and language in humans. In males, the development of the Broca area could last longer (DeCasien et al., 2020). The presence of a detectable and quantifiable cytoarchitectonic asymmetry does not necessarily point toward a functional implication in language *per se*. In fact, there seems to be no correlation between a specific cell type, or any relative cellular parameter/factor and linguistic functions, unlike the case of magnopyramidal neurons in the motor and premotor cortices, or the stria of Gennari in the visual cortex (Keller et al., 2009b). There seems to be however, definite differences between primary cortices (primary motor and association cortices) including Broca’s area, in terms of dendritic growth (Jacobs et al., 1993), which point out to higher functions. In that context, small round and dense neurons (presumably granule cells/interneurons) may indicate increased thalamic input on the left cortical layer 4, a feature albeit shared with several areas of the isocortex, compatible also with multiple commissural connections, which would point out toward Broca’s “complex” rather than a definite area (Hagoort, 2005), highly interconnected with neighboring areas (Anwander et al., 2007; Schmahmann et al., 2007).

Anatomical asymmetries and hemispheric specialization in both human and non-human primate brains are still subject to debate (Hopkins and Vauclair, 2011) and techniques are still being developed (Toga and Thompson, 2003), and usually rely on background/foreground segmentation such as for the neuropil in Spocter et al. (2012). Our technique does not use randomly scattered ROI boxes with black and white masking (Spocter et al., 2012), but rather local varying thresholding and Gaussian blur to determine cell bodies limits, and subsequently measure their features. Another type of promising technique for comparative neuroanatomy analysis is receptor mapping (Zilles and Palomero-Gallagher, 2017), in particular in the Broca area (Amunts et al., 2010). Using high resolution images, our flexible segmentation on cell populations by thousands permit an estimation of the form and area of a large amount of cells (Grisan et al., 2018), which elaborate techniques such as that of Amunts et al. (2010) cannot reach. Therefore, the scope of the present methodology can only be to precise cellular features from subsets of cortical neurons, such as segmented cortical layers.

The precise implications of such an asymmetry and cytoarchitecture in the brain of *Pan troglodytes*, and the functional relationship of this structural organizational peculiarity to language, require further functional studies.

## Data Availability Statement

The raw data supporting the conclusions of this article will be made available by the authors, without undue reservation.

## Ethics Statement

Ethical review and approval was not required for the animal study because the apes were brought to the Department of Comparative Biomedicine and Food Science of the University of Padua for post-mortem examination. Causes of death were unrelated to the nervous system and macro- and microscopic examination of the brain did not identify lesions or anomalies.

## Author Contributions

BC, CC, and J-MG designed the study. CC, EZ, and J-MG acquired the data. EZ, J-MG, and LC analyzed the data. EZ and J-MG drafted the manuscript. AG, AP, BC, CC, LC, EZ, and J-MG revised the manuscript. All the authors contributed to the article and approved the submitted version.

## Conflict of Interest

The authors declare that the research was conducted in the absence of any commercial or financial relationships that could be construed as a potential conflict of interest.
